# Floral biology, breeding system and pollination ecology of an endangered tree *Tetracentron sinense* Oliv. (Trochodendraceae)

**DOI:** 10.1186/1999-3110-54-50

**Published:** 2013-10-29

**Authors:** Xiaohong Gan, Lingling Cao, Xia Zhang, Huaichun Li

**Affiliations:** 1 College of Life Science, China West Normal University, Nanchong, 637009 PR. China; 2 Key Laboratory of Southwest China Wildlife Resources Conservation (Ministry of Education), China West Normal University, Nanchong, 637009 PR. China; 3 Library of China West Normal University, Nanchong, 637009 PR. China

**Keywords:** Floral biology, Breeding system, Pollination, *Tetracentron sinense* Oliv, Natural regeneration

## Abstract

**Background:**

*Tetracentron sinense* Oliv. is an endangered tree mainly distributed in south-central China. The breeding system and pollination ecology of *T. sinense* are unclear. With a conservation perspective, the floral biology, breeding system and pollination ecology of *Tetracentron sinense* Oliv. were investigated, in order to discuss the endangered factors related to pollination, and to provide important information for its conservation.

**Results:**

Our results revealed four important aspects of the reproductive biology of *T. sinense*. 1) *T. sinense* usually flowers by the beginning of June, and the flowering period of the population is about two months, and the florescence of florets lasted for 15 to 24 days with delicate fragrance. 2) The pollen/ovule ratio is 720 ± 28, and the outcrossing index is three. Artificial pollination experiments showed that *T. sinense* is self-compatible, with facultative xenogamy and no indication of agamospermy. 3) The pollination syndrome is ambophily, and self-pollination plays an important role in fruit production if wind and insect pollination is unavailable. 4) Insect pollinators were predominantly represented by Coleoptera, Diptera and Hymenoptera. Syrphid fly and bees were the main effective pollinators.

**Conclusions:**

The results suggested that *T. sinense* exhibits a mixed-mating system, and autogamy in its breeding system may provide reproductive assurance for the population maintenance. During flowering and pollination in natural population, the decrease of population density and harsh environmental condition might be one of crucial reasons resulting in endanger for this species.

**Electronic supplementary material:**

The online version of this article (doi:10.1186/1999-3110-54-50) contains supplementary material, which is available to authorized users.

## Background

Information on the reproductive biology of endangered plants is crucial for predicting their survival capacity and suggesting the appropriate conservation measures (Rodriguez-Perez^2005^). Such studies may help to identify the factors affecting the reproduction of individual plants and the subsequent maintenance or regeneration of populations. A number of studies have suggested that the maintenance of plant populations is strongly influenced by both demographic (reproductive success) and genetic (inbreeding depression, evolutionary potential) mechanisms (Frankham & Ralls^1998^, Saccheri et al.^1998^). As determining the demographic and genetic crucial parameters, the breeding system lies at the very heart of population health and maintenance (Gaudeul & Till-Bottraud^2004^).

Pollination is an important link of successful plant reproduction, and is often dependent on mutualistic interactions with animals. A reduction in pollinator service can directly influence reproductive output, decreasing the quantity and/or quality of fruit and seed set and promoting selfing in self-compatible species (Rodriguez-Perez^2005^). In a broad sense, an increase in selfing rate would result in a reduction in gene flow within and/or among populations and thus cause an increase in inbreeding depression (Buza et al.^2000^). Many studies have suggested that rare plants exhibit slightly higher levels of self-compatibility than common plants (Saunders and Sedonia^2006^). Therefore, the inbreeding depression of a rare plant due to a lack of pollinator visits, is usually the most important factor resulting in endanger for the species.

*Tetracentron sinense* Oliv. is a deciduous tree with a catkin-like inflorescence, and placed in its own family, Tetracentraceae previously (Fu & Bartholomew^2001^), or more commonly in Trochodendraceae together with the monotypic Trochodendron (Martyn & Peter^2007^). This species is today mainly distributed in south-central China (Fu & Bartholomew^2001^), and scattered within a region 1100–3 500 m above sea level (Martyn & Peter^2007^). Due to its rarity and poor ability to regenerate naturally, it is considered 'rare’ according to the International Union for the Conservation of Nature (IUCN) (Fu^1992^). Since its vesselless wood is quite rare in angiosperm suggesting primitiveness, the species has received much taxonomic attention (Chen et al.^2007^, Angiosperm Phylogeny Group^2009^). However, studies on the reproductive biology of *T. sinense* are relatively scarce (Gan et al.^2012^). Until recently, little is known about the breeding system and pollination ecology of *T. sinense*, the most well known plants due to its economic importance.

In this paper, floral biology, breeding system and pollination ecology of an endangered tree *T. sinense* Oliv. were studied with a conservation perspective. The objective of this work was to deepen the understanding of the reproductive biology of *T. sinense*, and to identify the factors resulting in endanger during flowering and pollination, as well as to examine the implications of the results for its conservation.

## Methods

### Plant material and study site

*Tetracentron sinense* Oliv. is a deciduous tree to 40 m or more tall, with arching branches. Twigs are greyish-brown, with leaves alternate on the current shoot. Inflorescences are short pedunculate, with 80–125 sessile flowers. Flowers are yellowish green and hermaphrodite. Sepals 4 and are ovate-orbicular, but petals are absent. Stamens 4 and are exserted at anthesis, and then the filaments are subterete or slightly flattened. Anthers are dehiscent by a lateral slit. Styles 4 and are erect at first, and then become recurved and subulate at anthesis. Stigmas are along the ventral surface of the style.

The studies were conducted between April 2010 and October 2011 at the Longwo conservation station of Dafengding Natural Reserve, located in the northeast Meigu County, Sichuan Province, China (103°08′E, 28°46′N). The study site has an annual mean temperature of 9.6°C and received an annual mean rainfall of 1100 mm, and the relative humidity about 85%, and the pH of soil is acidic. The rainy season is from late June to October. The conservation station comprises a large population of *T. sinense*, with more than one hundred individuals scattered across from 2000 to 2400 meters above sea level, and the average inter-tree distance is 15 ± 6 m.

### Phenology and floral biology

The time of anthesis initiation, and the blooming peak (months in which more than 50% of the individuals were in flower) and the termination in flowers were observed according to the methodology given in Dafni (1992). To describe the flowering period of an inflorescence and single flower, 40 inflorescences (4 inflorescences per tree) were tagged to study the time of anthesis additionally.

The floral biology was studied in 20 flowers from 10 inflorescences (one inflorescence per tree), revealing details such as morphological changes, anther dehiscence, nectar production, odor, and stigmatic status. For each flower, the pollen grains produced by two undehisced anthers were counted, and the average multiplied by the number of stamens. The ovules were counted after the dissection of ovaries under a stereoscopic microscope. The pollen–ovule ratio (P/O) and the outcrossing index (OCI) were calculated as recommended by Cruden (1977).

### Pollen viability and stigma receptivity

Two flowers in different flowering stage were respectively sampled at random. The pollen viability was detected by MTT assay, and deeply/completely stained pollens were considered viable, according to Dafni (1992). The stigma receptivity was verified with the catalase activity method (Zeisler^1938^).

### Breeding system

To determine the breeding system, pollination experiments were performed on randomly chosen from 10 trees in the population. On each plant, 4 to 10 inflorescences (depending on availability on each plant) were randomly tagged and only one of the treatments assigned to each inflorescence (i.e. to every single flower on that inflorescence). Inflorescences (n = 6 for each treatment) subjected to hand-pollination were bagged with thick waterproof paper bags before anthesis and bagged again after hand-pollination. The pollination bags were partly opened 5 days after each treatment to monitor fruit set. When required, the flowers were emasculated on the day of anthesis, before the anthers dehisced. The following treatments were conducted (Saunders and Sedonia,^2006^): (1) Control: the flowers were tagged and left to open pollination; (2) Geitonogamy: emasculated and bagged flowers were hand-pollinated using pollen from the same plant but from another flower (to test for self-compatibility); (3) Xenogamy: emasculated and bagged flowers were hand-pollinated using pollen from different plants (to test for outcrossing ability); (4) Cross-pollination under natural conditions: uncovered and emasculated flowers were pollinated freely (to test for pollen limitation); (5) Autogamy: flower buds were bagged and isolated from visitors (to test for autonomous self-pollination within a single flower); (6) Apomixis: flowers were bagged and emasculated before anthesis (to test for the agamospermy).

In addition, 1000 seeds for each treatment were weighed to determine the mean seed mass. To look for differences in germination among pollination treatments, a germination experiment using 100 randomly chosen seeds from each treatment was performed according to Luo et al. (2010).

### Pollination

Flower visitors were observed in the field over the peak of flowering in 2010 and 2011 on sunny days. Observations were made synchronously by several observers posted at three sites in the population from 0800 to 1800 every day, and were set periods (40 min). The pollinators and their behavior during the visits were observed and recorded, and used in conjunction with descriptions of floral morphology to evaluate their efficacy as pollinators. Representative floral visitors were collected and identified by specialists, and some of them were observed and photographed under a scanning electron microscopy for the presence and placement of *T. sinense* pollen (Saunders and Sedonia^2006^). These data, along with field observations, were used to ascertain which visitors were likely to be pollinators. *T. sinense* pollen was sufficiently distinct from synchronously flowering species that it could be distinguished on the insects with confidence. Voucher specimens of the insect visitors are deposited in the Herbarium of China West Normal University. Nearly 270 h of observation at the reserve was spent in the field watching flowers for floral visitors.

To determine the role of wind in pollination, glass microslides coated in sticky vaseline gel were tied vertically at 80 cm height in the canopy, and 2, 4, 6 and 8 m away from canopy of the isolated trees (n = 5) and scored after 24 h (Dafni^1992^). In addition, previously emasculated flowers (n = 200) were bagged with 1 mm^2^ plastic mesh to prevent the entry of insects and then monitored for fruit set. Some of these bagged flowers (n = 20) were removed after 24 h to determine the number of pollen grains deposited on the stigma as a result of wind.

### Statistical analyses

The fruit setting, seed mass and germination rate among pollination treatments in artificial pollination experiment were analyzed with one-way ANOVAs followed by Duncan test using the statistic software SPSS 17.0.

## Results

### Phenology and floral biology

Flower buds appeared at the end of April in this population. Flowering started by the beginning of June, and reached its maximum by the beginning of July, and finished at the beginning of August. Fruiting started 4 weeks after the commencement of blooming. Fruits became mature by the beginning of October. Thus, the flowering period of the population lasted for about two months, and fruiting period for about 3 months.

The green catkin-like inflorescences were produced near the base of the petiole, and sessile flowers with absent petals were laid in whorls of 4. The flowering period of an inflorescence lasted for about one month, and the lifespan of a single flower (from sepals opening to stamens lost) was 15–24 days. At the early stage of anthesis, the delicate fragrance from *T. sinense* flower was investigated, and then gradually weakened. The nectary is on the surface of ovary, and some flower-visiting insects were observed to stretch their mouthparts into the floral base for nectar.

**Figure 1:**
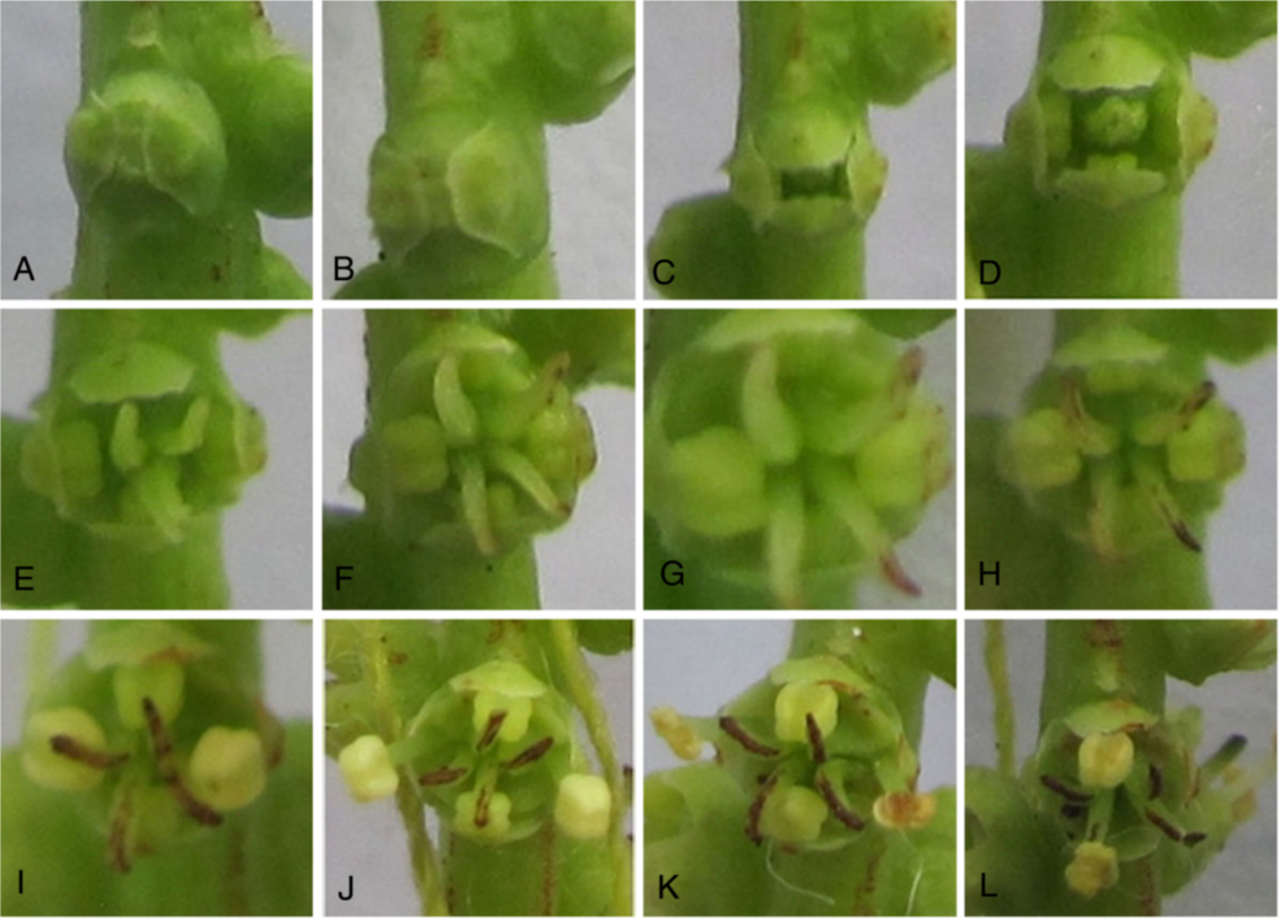
**Flowering dynamics of** ***T. sinense*** **. A-B**: flower buds with closed sepals; **C**: sepals gradually opened to form a rectangular split; **D**: stamens on the right and left of the flower were showing, and four green styles gathered together and became erect; **E**: the styles gradually elongated and became recurved; **F-G**: the stamens began to elongate with the elongation of the styles, and gradually became greenish yellow; **H**: the styles reflexed to an angle of 90˚ when its color was light brown; **I-J**: the stamens on the right and left of the flowers finished elongation and were positioned higher than the stigmas, and then their anther dehisced; **K**: the stamens on the upper and lower elongated gradually; **L**: the stamens on the upper and lower were positioned higher than stigmas, and then dehisced.

**Figure 2:**
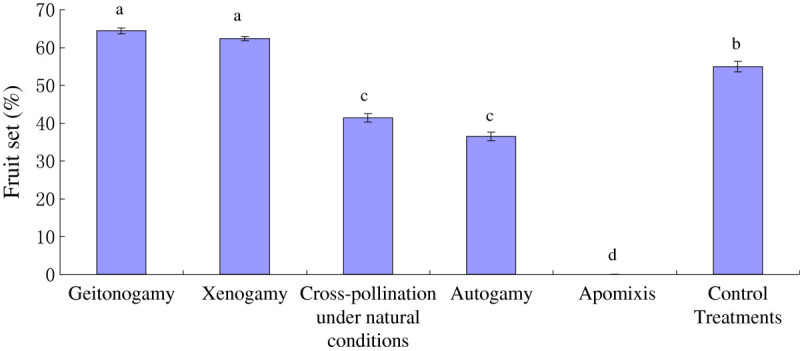
**The fruit set of** ***Tetracentron sinense*** **under different treatments.**

**Figure 3:**
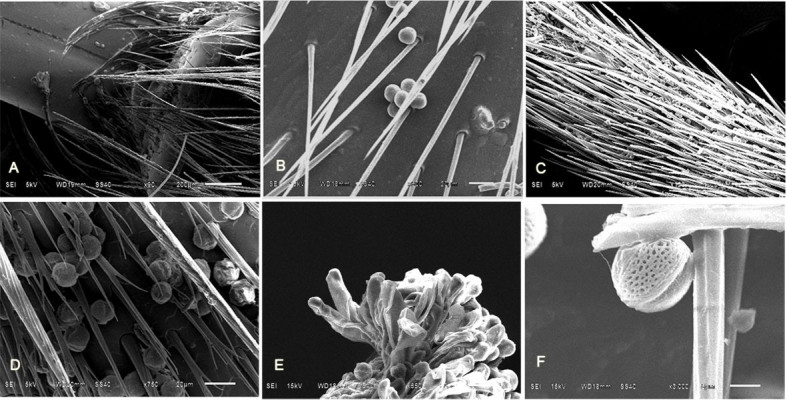
**The SEM observation of insects bodies, pistil and pollen of** ***T. sinense*** **.** **A**: showing the pollens on the mouthparts; **B**: showing the pollens on the chest; **C-D**: showing the pollens on the legs; **E**: the pistil of T. sinense, arrow showing the pollen; **F**: the pollen of T. sinense.

**Figure 4:**
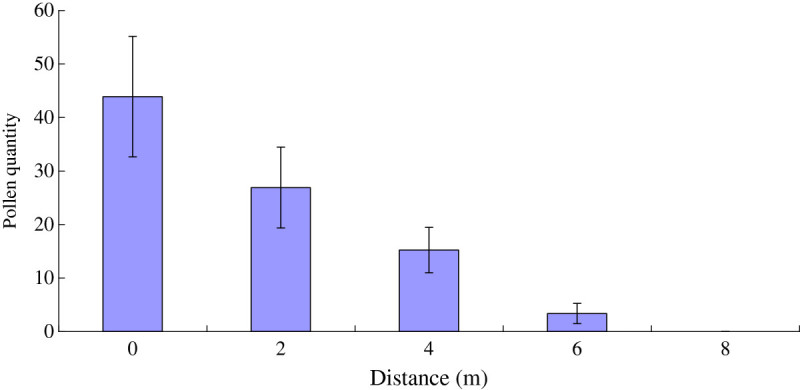
**Pollen quantity of** ***T. sinense*** **in different distances from the pollen source.**

The flowering dynamic of a single flower could be divided into the following stages: (1) Flower buds with closed sepals(Figure 1A-B); (2) Sepals opening: sepals gradually opened to form a rectangular split, leading to the emergence of erect green styles and stamens from the sepals (Figure 1C-D); (3) Style elongating: styles gradually separated from each other and elongated, when the stamens became greenish yellow (Figure 1E-G); (4) Style reflexing: styles gradually became recurved to an angle of 90˚, when its color was light brown(Figure 1H); (5) the stamens on the right and left of the flowers elongating: these stamens became yellow, and their filaments elongated gradually until higher than stigma (Figure 1I); (6) the anthers on the right and left of the flowers dehiscing: the anthers dehisced by a lateral slit, when the stigmas became darken (Figure 1J); (7) the stamens on the upper and lower of flowers elongating: the stamens began to elongation after the bilateral anthers dehisced (Figure 1K); (8) the anthers on the upper and lower of flowers dehiscing: the stamens were positioned higher than the brown stigmas, and then their anthers dehisced( Figure 1L); (9) The termination: stamens gradually wilted and fell, but sepals and styles would be persistent.

### Timing of pollen viability and stigma receptivity

Percent of viable pollen from un-dehiscent anther was about 32.65 ± 1.91%, when the stigma receptivity is higher (Table 1), indicating the flowers of *T. sinense* are protogynous. With the development of a single flower, pollen viability and stigma receptivity would increase. As the stamens on the right and left of the flowers were mature and dehisced, the pollen viability and the stigma receptivity were all the highest. Whereafter, pollen viability and stigma receptivity decreased gradually until flowers wilted.

**Table 1:** **The pollen viability and stigma receptivity of** ***T. sinense***

Developmental stage of flower	Pollen viability/%	Stigma receptivity
Sepal opening	32.65 ± 1.91	++/--
Stigma reflexing	30.63 ± 1.70	+++/-
Stamens on the right and left of flower elongating	36.44 ± 2.71	+++/-
Dehiscence of anthers on the right and left	62.47 ± 1.89	++++
Stamens on the upper and lower of flower elongating	55.35 ± 2.64	+++/-
Dehiscence of anthers on the upper and lower	47.02 ± 2.32	++/- -

Note: '++++’ showing the stigma receptivity is the highest; '+++/-’ showing the receptivity of some stigmas is stronger and another is weaker; '++/--’ showing some stigmas have receptivity while another have no receptivity.

### Pollen–ovule ratio (P/O) and the outcrossing index (OCI)

In a single flower of *T. sinense*, the pollen number is 18000 ± 706 (n = 20), and the ovule number is 25 ± 1 (n = 20), so the pollen-ovule ratio is 720 ± 28.

The outcrossing index (OCI) was three. This has been calculated by summing the following values:

1. Diameter of inflorescence is 4.02 ±0.15 mm (n = 10) = 2;
2. Temporal separation between anther dehiscence and stigma receptivity: protogyny = 0; and
3. Stigma and anthers spatially separated: stamens are positioned higher than stigmas (stamens on the right and left of flower 1.82 ± 0.05 mm; stamens on the upper and lower of flower 0.86 ± 0.02 mm, n = 20) = 1.

### Breeding system

Fruits were produced after the treatments of geitonogamy and xenogamy (Figure 2), indicating that *T. sinense* is outcrossing fertile and self-compatible. The treatment of autogamy resulted in fruit set (36.45 ± 1.10%), indicating the automatic self-pollination within a single flower of *T. sinense*. No fruits were obtained in the emasculated and bagged flowers, which fell soon after the treatment, indicating the absence of agamospermy for this species. There was significant difference (one-way ANOVA, F = 104.438, P < 0.001) in the probability of setting fruit between xenogamy (62.32 ± 0.57%) and control (54.88 ± 1.41%) or cross-pollination under natural conditions (41.37 ± 1.16%), showing that the fruit set of *T. sinense* in the natural population was limited by pollinator.

In addition, seeds from control and autogamy treatments were significantly lighter (one-way ANOVA, F = 11.269, P < 0.001) than that from xenogamy treatment (Table 2). The germination rate of seeds from xenogamy and control treatment was significantly higher than that from autogamy treatments.

**Table 2:** **The weight and germination rate of seed for** ***T. sinense*** **from three pollination treatments**

Treatment	Xenogamy	Autogamy	Control
Weight per 1000 seeds (mg)	90.70 ± 2.20a	76.80 ± 1.50b	81.60 ± 0.80b
Germination rate of seed (%)	75.69a	19.26c	51.51b

Note: Error bars are 95% confidence intervals for the mean. Different letters indicates significant differences between pollination treatments (*P* < 0.001).

### Pollination

A total of 105 40-min observation/collection sessions were conducted in this population. Several orders of insects visited *T. sinense* flowers, including Coleoptera (beetles: Cerambycidae), Diptera (Syrphid fly: Syrphidae) and Hymenoptera (bees: Apidae and Vespidae) (Table 3). Syrphid fly and bees were most prevalent on calm, warm days during the mid- to late-afternoon hours.

**Table 3:** **Floral visitors and their rewards**

Order	Family	Species	Reward
Coleoptera	Cerambycidae	*Parastrangalis* sp.	Nectar
		*Pidonia* sp.	Nectar
Diptera	Syrphidae	*Eristalis tenax* Linnaeus	Pollen, nectar
		*Syrphus vitripennis* Meigen	Pollen, nectar
		*Allograpta nigritibia* Huo	Pollen, nectar
		*Cheilosia* sp.	Pollen, nectar
		*Palumbia sinensis* Curran	Pollen, nectar
		*Sphaerophoria sp.*	Pollen, nectar
Hymenoptera	Apidae	*Xylocopa sinensis* Smith	Pollen, nectar
		*Haborpoda* sp.	Pollen, nectar
	Vespidae	*Vespa* sp.	Pollen, nectar

The potential of floral visitors was evaluated to serve as pollinators by the presence of *T. sinense* pollen on their bodies (such as mouthparts, chest and legs) (Figure 3). The insects of Syrphid fly and bees had the pollen of *T. sinense* on their mouthparts, and the length of mouthparts was sufficient to effectively transfer pollen to the stigma of individual flowers. The species of beetles displayed nectaring behavior on the flowers, but none of the collected specimens had *T. sinense* pollen on their bodies. Thus, it appears that the species of Cerambycidae visited *T. sinense* primarily as a nectar source, and were not considered to be potential pollinators.

These insect visitors exhibited two kinds of different behavioral patterns. The species of Cerambycidae and Syrphidae often stayed on an inflorescence for about 1 min, when crawling and stretching their mouthparts into the flower base for foraging nectar. In contrast, the insects of Vespidae and Apidae usually flew around the inflorescence, and only stayed briefly to stretch their mouthparts into the flower base for foraging nectar, and then quickly flew to another inflorescence.

The visiting behavior of potential pollinators was significantly influenced by the external environmental factors, such as temperature (p = 0.019). On sunny days, the temperature was lower during the period of 0900 to 1040, when the visiting insects were rarely observed (7.13 ± 0.77 times). The visiting frequency of insects increased with the increase of temperature and illumination, and reached its peak (18.33 ± 3.76 times) from 1100 to 1340. Thereafter, the number of visiting insects reduced rapidly due to the decrease of temperature and illumination. Especially, almost no pollinator was observed after 1540 on sunny days, as well as on cloudy or rainy days.

Experiments (n = 100) conducted to determine the efficacy of wind pollination. The result showed 2.5 ± 0.7% fruit set. The density of airborne pollen grains in the canopy was 43.88 ± 11.26 cm^-2^ and it declined completely 8 m away from the canopy (Figure 4).

## Discussion

### The relationship between flowering and pollination

Our results suggest that the pollination syndrome of *T. sinense* is ambophily (pollination by both wind and insects). In pollination experiments, emasculated flowers bagged with plastic mesh set fruits, indicating *T. sinense* is wind-pollinated. Because inflorescences covered by paper bags did not set fruit, but did when hand-pollinated, the tree does not set fruits by apomixis. A lot of airborne pollen caught on the glass microslides also supports the effectiveness of wind pollination. The anemophilous floral features of *T. sinense* include the absence of petals, the papillate stigma, exposed anthers and stigma and elongated catkin-like inflorescences (Vikas^2011^; Yamasaki & Sakai^2013^). This species also appears to be insect-pollinated because insects with pollen on their bodies visited the flowers. As most of the pollen was observed on the mouthparts, chest and legs of flower visitors (Syrphid fly and bees), these insects may be effective pollinators. *T. sinense* exhibits floral characteristics that are adapted to insect pollination: (1) the floral traits such as herkogamy, protogyny and the recurved styles, are all helpful for insect-pollination (Mayer et al.^2011^); (2) The inflorescences with many closely ranked and sessile flowers are beneficial for insect visitors to stay for a long time; (3) The anthesis of a single flower is longer (lasted for 15 to 24 days), and the time of releasing pollen for the stamens of *T. sinense* flower is relatively concentrated; (4) In the population, the blossom is relatively concentrated in July, and the delicate fragrance and nectary were investigated during anthesis, which can provide important visiting reward as pollen and nectar for insect visitors (Dafni^1992^, Yamasaki & Sakai^2013^).

The extent of ambophily in angiosperms has been recently reviewed by Culley et al. (2002). Relatively few flowering plants show ambophily (pollination by both wind and insects) (Yamasaki & Sakai^2013^). Generally, ambophily is considered an intermediate condition during a transition to either full wind pollination or biotic pollination (Culley et al.^2002^). In some pioneer plants (including *Salix* spp. and *Azadirachta indica*), ambophily is advantageous because of changing wind conditions during forest succession (Karrenberg et al.^2002^, Vikas^2011^). In other plants (for example, *Mallotus japonicus*), the decrease of population density also contribute to the maintenance of ambophily (Yamasaki & Sakai^2013^). Previous studies have reported that in wind-pollinated plants, pollen limitation increases rapidly with the increases in distance from a pollen source (Hesse & Pannell^2011^). In contrast, pollen limitation does not strongly depend on distance from a pollen source in insect-pollinated plants (Albrecht *et al.*^2009^). As been extensively deforested in history resulting in the decrease of its population density, at present most *T. sinense* sporadiclly scatter in the mountains, valleys, stream or steep cliffs (Fu^1992^). In the population, the distance between individuals is 15 ± 6 m, larger than the average traveling distance (8 m) of airborne pollens, and the amount of airborne pollen rapidly decreased with distance from the pollen source, resulting in the increase of pollen limitation from wind pollination. Therefore, the entomophilous characteristic of *T. sinense* may remedy the pollen limitation from wind pollination, which is an adaptive strategy for *T. sinense* to assure the reproduction success. So we think the maintenance of ambophily in *T. sinense* should attribute to the decrease of population density.

In this study, fruits were produced after the autogamy treaments, suggesting *T. sinense* is autonomously self-pollinated. *T. sinense* also exhibits several floral traits specially associated with self-pollination: bisexual flower, the recurved style positioned lower than the anthers, protogyny, no temporal separation between pollen viability and stigma receptivity (Faegri & van der Pijl^1979^). However, the fruit set, seed mass and germinate rate from autogamy are significantly lower than that from xenogamy and control. In addition, pollination experiments showed that fruit set of *T. sinense* in natural population was limited by pollinators, and there is no agamospermy. So we conclude that autonomous self-pollination plays an important role in fruit production if wind- and insect-pollination is unavailable.

### Breeding system and its adaptability

Artificial pollination experiments showed that *T. sinense* is self-compatible and outcrossing fertile, and no apomixes is occurred. The fruit set from xenogamy is higher than that from control and cross-pollination under natural conditions, indicating the pollinator limitation in natural population. The results showed that *T. sinense* is self-compatible, and sometimes needs pollination vector, and can be pollinated by wind and insect (Syrphid fly and bees), which is in accordance with that from out-crossing index and pollen-ovule ratio. According to Dafni (1992), the out-crossing index is 3, indicating *T. sinense* is self-compatible, with facultative xenogamy, and sometimes needs pollinators. The pollen-ovule ratio (720 ± 28) belongs to the range of 244.7-2588.0 corresponding to the values reported for facultative xenogamy (Cruden^1977^). Therefore, the mating system of *T. sinense* is mixed with self-pollination and out-crossing, and there is no apomixes. While, the fruit set from xenogamy and control is respectively higher 25.87% and 18.43% than that from autogamy, and the seed germination rate from autogamy is respectively lower 56.43% and 32.35% than that from xenogamy and control, suggesting that outcrossing predominated in the breeding system of *T. sinense.*

Evolution of plant mating strategies always has one main theme related to the evolution of cross- versus self-fertilization (Barrett^1998^). The evolutionary pathway from obligate outcrossing based on self-incompatibility to predominant self-fertilization has possibly been followed by more different lines of evolution in flowering plants than any others (Stebbins^1974^). It was predicted that predominant selfing and predominant outcrossing should be alternative stable outcomes of mating system evolution in most plant population (Lande and Schemske^1985^), which was be validated by the existence of some species with stable, mixed mating-systems in natural plant populations (Barrett and Harder^1996^). Our results showed that in the mating system of *T. sinense* existed both selfing and outcrossing simultaneously. However outcrossing predominated in *T. sinense*, and autonomous self-pollination just played an assistant role to assure production in the breeding system, when conditions for outcrossing are unfavorable, such as shift of environment conditions, or the normal pollinators are missing or reduced in abundance (Huang et al.^2006^).

### Factors resulting in endanger during flowering and pollination

Despite the occurrence of successful insect pollination, the pollinator activity is easily influenced by environmental condition, and almost no visiting insect was observed on rainy or cloudy days in this study. In Meigu Dafengding Natural Reserve, the flowering period of *T. sinense* is just coincident with the rainy season (June to August), which has a negative impact on the flower-visiting behavior of insects. In addition, the density of airborne pollen grains declined completely 8 m away from the canopy, and the average inter-tree distance is 15 ± 6 m due to the decrease of population density, which may have a negative effect on wind-pollination for this species. Thus, the successful reproduction for *T. sinense* may only rely on self-pollination or geitonogamy in most cases, which would result in autogamous and geitonogamous selfing. However, selfing would result in inbreeding depression or reduction of genetic diversity, and then decreasing the ability of the population to adapt in response to environmental change (Buza et al.^2000^, Gaudeul & Till-Bottraud^2003^), which would be unfavorable to natural regeneration of *T. sinense.* Thereby, the decrease of population density and harsh environmental condition during flowering and pollination might be one of crucial reasons resulting in endanger for this species. The urgent task is to comprehensively analyze the factors resulting in poor ability to regenerate naturally during genetic and developmental process of *T. sinense*, and to formulate effective protective measures. Especially, it is necessary to enlarge its population and individual number as soon as possible through artificial intervention.

## Conclusion

In summary, we investigated the floral biology, breeding system and pollination ecology of *Tetracentron sinense* Oliv. The occurrence of successful wind and insect pollination indicates that *T. sinense* is ambophilous, and the maintenance of ambophily may attribute to the decrease of population density. The breeding system of *T. sinense* is mixed with self-pollination and out-crossing, with no indication of agamospermy. Outcrossing predominated in the mating system, and autonomous self-pollination in its breeding system may provide reproductive assurance for the population maintenance. During flowering and pollination in natural population of *Tetracentron sinense,* the decrease of population density and harsh environmental condition might be one of crucial reasons resulting in endanger for this species.

## Authors’ original submitted files for images

Below are the links to the authors’ original submitted files for images. Authors’ original file for figure 1 Authors’ original file for figure 2 Authors’ original file for figure 3 Authors’ original file for figure 4
