# Supplementary figures and images for: Floral biology, breeding system and pollination ecology of an endangered tree Tetracentron sinense Oliv. (Trochodendraceae)
Source: Bot Stud. 2013 Oct 29;54:50. doi: 10.1186/1999-3110-54-50 (PMC5430371; doi:10.1186/1999-3110-54-50)

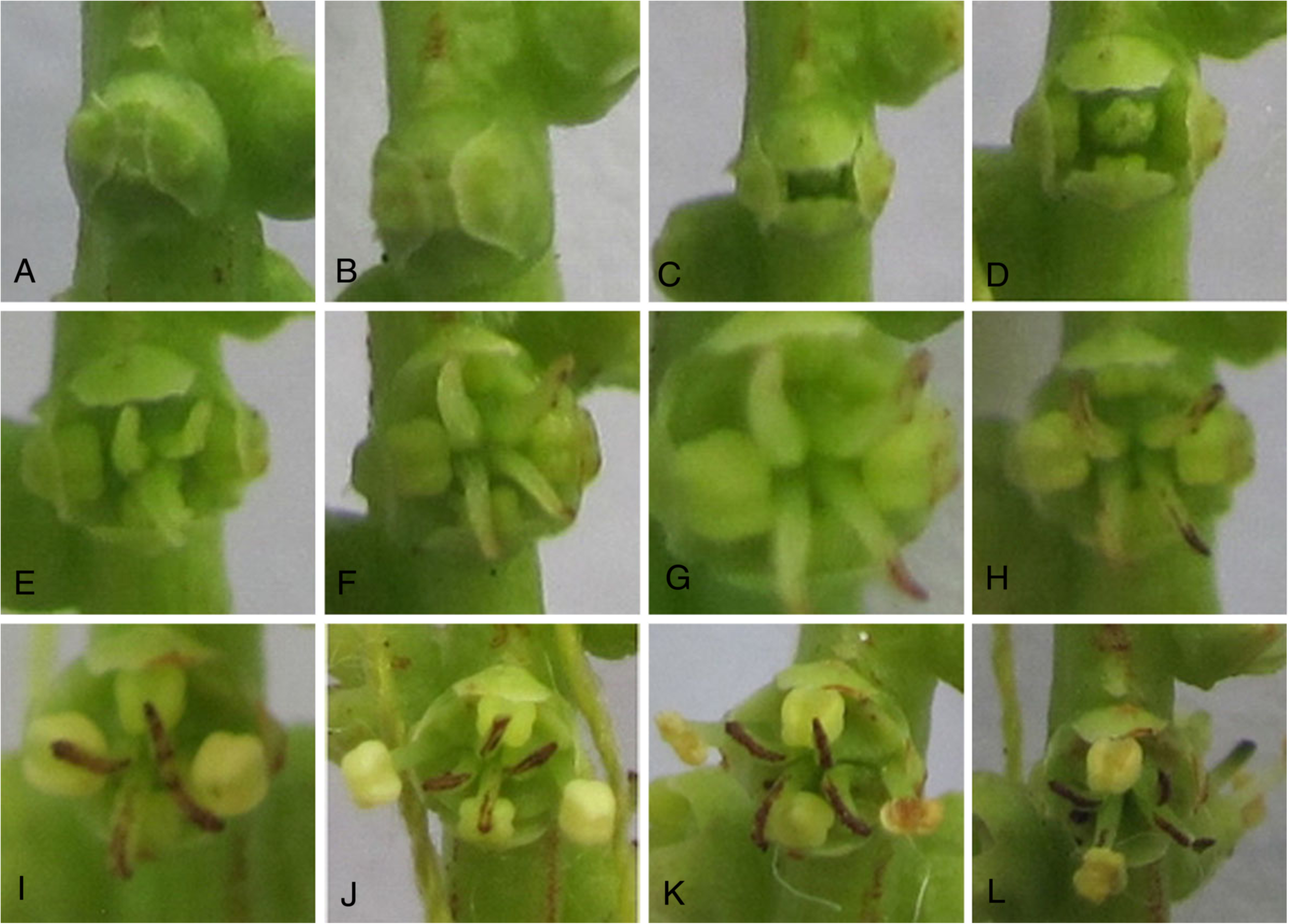

Supplement: Supplementary file 1 — Authors’ original file for figure 1 [file 40529_2013_42_MOESM1_ESM.tif]

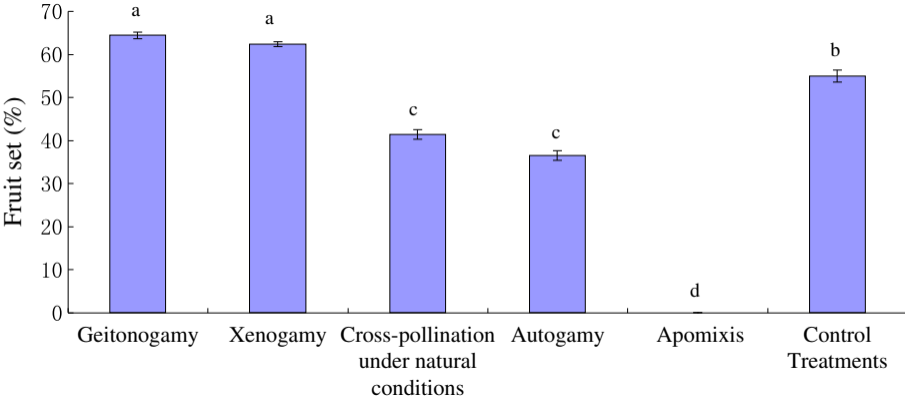

Supplement: Supplementary file 2 — Authors’ original file for figure 2 [file 40529_2013_42_MOESM2_ESM.pdf]

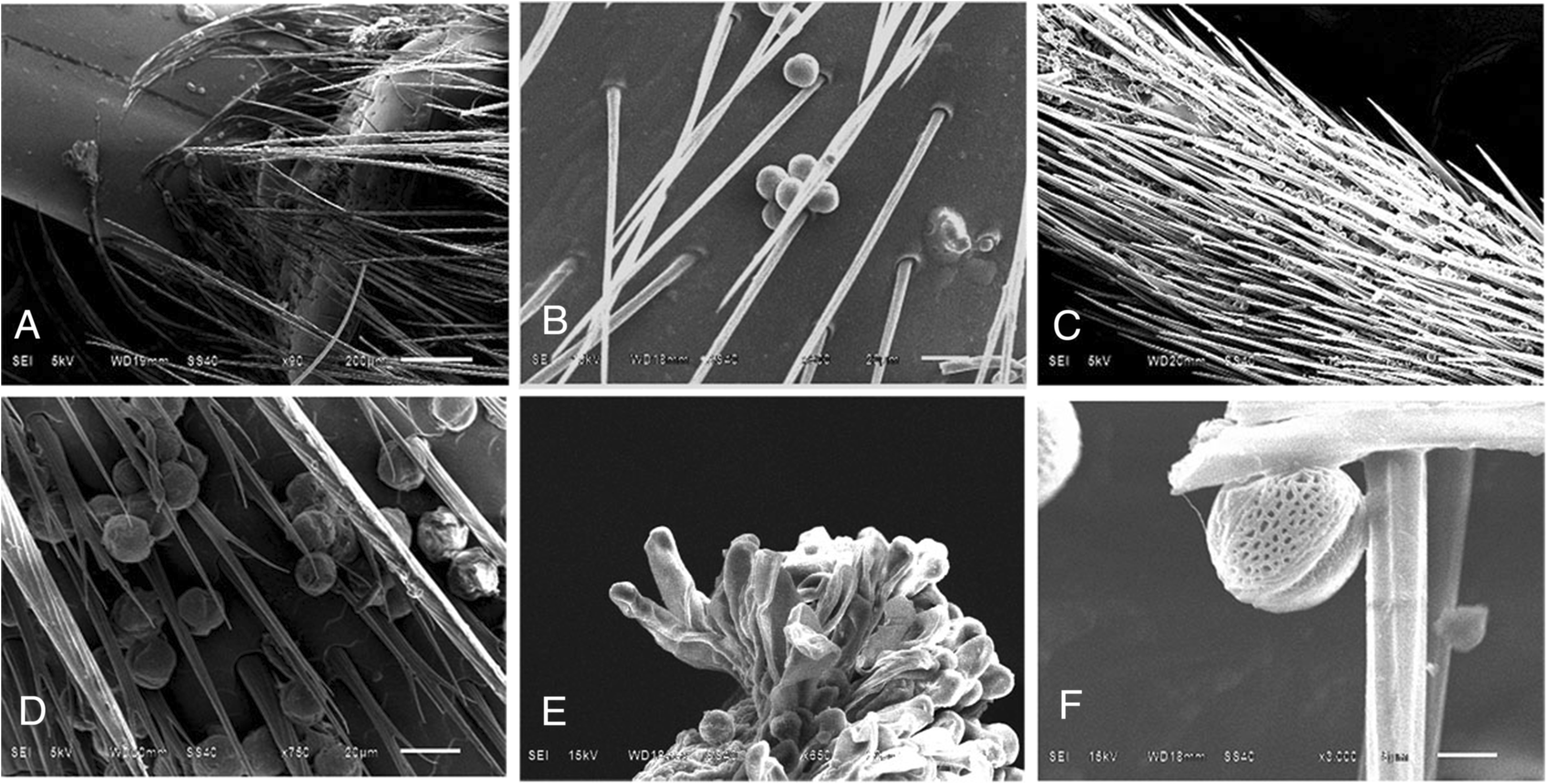

Supplement: Supplementary file 3 — Authors’ original file for figure 3 [file 40529_2013_42_MOESM3_ESM.tif]

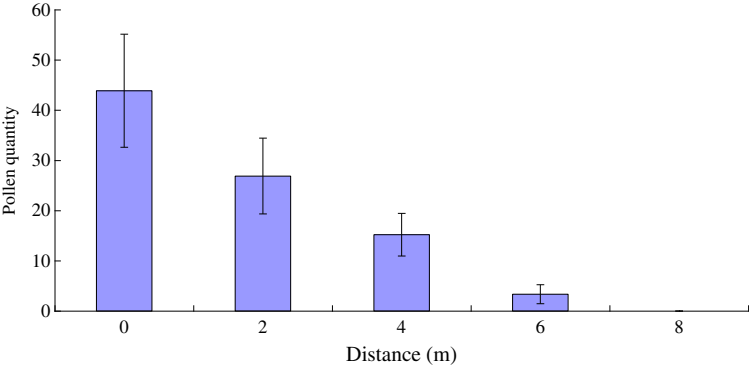

Supplement: Supplementary file 4 — Authors’ original file for figure 4 [file 40529_2013_42_MOESM4_ESM.pdf]
